# Body mass index increase: a risk factor for forced expiratory volume in 1 s decline for overweight and obese adults with asthma

**DOI:** 10.1183/23120541.00110-2022

**Published:** 2022-10-24

**Authors:** Nicolás Bermúdez Barón, Hannu Kankaanranta, Linnea Hedman, Martin Andersson, Caroline Stridsman, Anne Lindberg, Eva Rönmark, Helena Backman

**Affiliations:** 1 Dept of Public Health and Clinical Medicine, Section of Sustainable Health, OLIN unit, Umeå University, Umeå, Sweden; 2 Dept of Internal Medicine and Clinical Nutrition, Krefting Research Centre, Sahlgrenska Academy, Gothenburg University, Gothenburg, Sweden; 3 Faculty of Medicine and Health Technology, Tampere University Respiratory Research Group, Tampere University, Tampere, Finland; 4 Dept of Respiratory Medicine, Seinäjoki Central Hospital, Seinäjoki, Finland; 5 Dept of Public Health and Clinical Medicine, Section of Medicine, OLIN unit, Umeå University, Umeå, Sweden

## Abstract

**Background:**

With increasing prevalence of overweight and obesity, it is important to study how body mass index (BMI) change may affect lung function among subjects with asthma. There are few prospective studies on this topic, especially with separate analyses of those with normal and high BMI. The aim of the present study was to prospectively study the association between annual BMI change and annual lung function decline, separately among those with normal initial BMI and overweight/obesity, in an adult asthma cohort.

**Methods:**

A population-based adult asthma cohort was examined at study entry between 1986 and 2001 and at follow-up between 2012 and 2014 (n=945). Annual BMI change was analysed in association with annual decline in forced expiratory volume in 1 s (FEV_1_), forced vital capacity (FVC) and FEV_1_/FVC separately in those with normal weight (BMI 18.5–24.9) and overweight/obese subjects (BMI ≥25) at study entry. Regression models were used to adjust for sex, age, smoking, inhaled corticosteroids use and occupational exposure to gas, dust or fumes.

**Results:**

Overweight/obese subjects had lower FEV_1_ and FVC but slower annual FEV_1_ and FVC decline compared to those with normal weight. After adjustment through regression modelling, the association between BMI change with FEV_1_ and FVC decline remained significant for both BMI groups, but with stronger associations among the overweight/obese (FEV_1_ B_[Overweight/obese]_=−25 mL *versus* B_[normal weight]_=−15 mL). However, when including only those with BMI increase during follow-up, the associations remained significant among those with overweight/obesity, but not in the normal-weight group. No associations were seen for FEV_1_/FVC.

**Conclusions:**

BMI increase is associated with faster FEV_1_ and FVC decline among overweight and obese adults with asthma in comparison with their normal-weight counterparts.

## Abstract 2

**BMI increase is associated with faster FEV_1_ and FVC decline in adults with asthma, and this association is stronger among overweight and obese adults with asthma than among their normal-weight counterparts** https://bit.ly/3PDxOk0

## Introduction

Asthma is characterised by a variable expiratory airflow limitation which may become persistent [1] and lead to a more accelerated FEV_1_ decline [2]. Impaired lung function in asthma is known to be associated with multiple factors such as current smoking [3] and smoking pack-years [4] but also obesity [5]. Consequently, these factors relate to a worse asthma control [6] and severity [7], which in turn associate with lower quality of life [8–10].

Overweight and obesity are becoming more common worldwide [11], and increased body mass index (BMI ≥25) [12, 13] is known to be related to lower FEV_1_. There are cross-sectional studies that have shown that obese adults with asthma have lower FEV_1_ than adults with asthma having normal weight [5]. However, there are few population-based longitudinal studies on this topic [14], and specifically those focusing on adults with asthma have been lacking. One prospective study [15] showed that BMI increase indeed was related to FEV_1_ decline among young adults with non-obstructive asthma, but this study did not separate overweight and obese subjects from those with normal weight, and thus it is not known if the association differs depending on initial BMI.

Asthma treatment response has been shown to vary depending on the underlying asthma phenotypes and should ideally be tailored based on clinical and molecular traits [1, 16]. Despite the available pharmacological therapies, obese asthma patients often remain symptomatic, with more exacerbations and hospitalisations, although these results are less clear regarding overweight asthma patients [17–19]. Overweight and obese subjects with asthma are more commonly resistant to the standard treatment with inhaled corticosteroids (ICS), likely related to frequent lack of the eosinophilic airway inflammation and also to dysregulation of some metabolic signalling pathways [20–22]. Thus, non-pharmacological interventions such as weight loss and smoking cessation may be particularly important for this group [1, 3, 23, 24].

In summary, prospective studies focusing on the association between longitudinal changes in BMI and lung function in asthma are important but scarce [15], especially with sample sizes allowing for stratification by baseline BMI [25]. Thus, our aim is to study the association between annual BMI change and annual lung function decline, separately among those with normal weight and overweight/obesity, in a 10- to 28-year follow-up of a population-based adult asthma cohort.

## Methods

### Study design

This study is part of the Obstructive Lung Disease in Northern Sweden (OLIN) research programme. A population-based cohort of adults with asthma living in the northernmost county of Sweden, Norrbotten (n=2055, 55% women, aged 19–72 years) was identified from five original population-based cohorts during 1986–2001. The inclusion criteria were strictly predefined based on data from clinical examinations and detailed structured interviews.

Participants from cohorts I–IV with at least one of the following criteria were included:

1) Physician-diagnosed asthma or report of ever having asthma;
2) Asthmatic wheeze without a cold in the last 12 months in combination with attacks of shortness of breath/wheeze or use of asthma medication;
3) Attacks of shortness of breath in the last 12 months with FEV_1_ reversibility of both ≥12% and ≥200 mL; and
4) Attacks of shortness of breath or wheeze in the last 12 months with methacholine bronchial hyperresponsiveness.

All participants from cohort V were included as they were physician-diagnosed with adult-onset asthma from primary care and had a medical history of asthma together with methacholine bronchial hyperresponsiveness [26].

At study entry in 1986–2001, a detailed structured interview was performed including questions regarding asthma diagnosis, potential risk factors, treatment of obstructive respiratory diseases and occupation. Clinical examinations included spirometry and assessment of height and weight.

The follow-up was performed during 2012–2014 in which those still alive and living in the county (n=1425) were invited. In total, n=1006 (71%) participated in similar clinical examinations that included structured interviews, spirometry and measurement of height and weight [26].

Participants with BMI ≥18.5 at study entry and not missing FEV_1_ or BMI measurements both at study entry and at follow-up were included in the study sample (n=945) (supplementary figure S1).

### Lung function

At study entry, spirometry was performed with a Vicatest VCT-5 dry volume spirometer (Mijnhardt, Bunnik, The Netherlands) following the 1987/1994 American Thoracic Society (ATS) guidelines with a repeatability criterion of the two best measurements of ≤5% and ≤100 mL when the best FEV_1_ was ≤1 L [27, 28].

Spirometry at follow-up was performed with a pneumotach spirometer (Jaeger Masterscope, Hoechberg, Germany) following the 2005 European Respiratory Society (ERS)/ATS guidelines with a repeatability criterion of ≤150 mL [29].

FEV_1_ and FVC were measured (before bronchodilatation), expressed both in millilitres and as per cent of predicted (pp) using the OLIN reference values [30]. The ratio between FEV_1_ and FVC was also calculated. Post-bronchodilatory values were not included in the current study as they were available only in certain subsamples.

Changes in FEV_1_, FVC and FEV_1_/FVC were calculated as the value at follow-up minus the value at study entry. The annual decline was calculated as the change divided by the numbers of years between examinations (10–28 follow-up years), expressed in terms of pp per year and millilitres per year, respectively, as ΔFEV_1_pp/y, ΔFVCpp/y, ΔFEV_1_mL/y, ΔFVCmL/y and ΔFEV_1_/FVC/y.

### BMI

At study entry, participants were categorised based on BMI (kg·m^−2^) as normal weight (18.5≤BMI<25) and overweight/obese (BMI≥25). Additional analyses were also performed by dividing overweight/obese into the categories overweight (25≤BMI<30) and obese (BMI≥30); these results are presented in the supplementary material.

BMI change was calculated as the value at follow-up minus the value at study entry. The annual BMI change was calculated by dividing the BMI change with the number of years between examinations (ΔBMI/y). The sample was also divided by quartiles for ΔBMI/y with cut-offs of Q1<0.042 (n=236), 0.042≤Q2<0.142 (n=237), 0.142≤Q3<0.274 (n=236) and Q4≥0.274 (n=236).

### Other definitions

Data at study entry included sex, age, ICS use in the last 12 months, smoking habits (categorised as nonsmoker, ex-smoker and current smoker) and original cohort (I–V).

Follow-up data included smoking pack-years, which was calculated by multiplying the number of packs of cigarettes smoked per day by the number of years of smoking, as well as ICS use in the last 12 months. Occupational exposure to gas, dust or fumes (GDF) at follow-up was defined by the question “Have you been heavily exposed to dust, gases or fumes at your work (not including tobacco)?”.

Changes in smoking habits from study entry to follow-up were defined as: never-smokers (nonsmokers on both occasions), ex-smokers (from nonsmokers or ex-smokers to ex-smokers), quitters (from current smokers to ex-smokers), current smokers (from nonsmokers or ex-smokers or current smokers to current smokers) and inconsistent (from ex-smokers or current smokers to nonsmokers).

### Statistical analyses

The analyses were made with IBM Statistical Package for the Social Sciences (SPSS) software version 26. Results were stratified by BMI categories (normal and overweight/obese) at study entry. The assumption of normal distributions for annual changes in BMI and lung function were assessed by histograms. Spearman correlation coefficients (rho) were used to evaluate correlations between annual changes in BMI and lung function. Comparisons of proportions of female sex, smoking categories and ICS use across BMI categories were done by Chi-squared test. Comparisons of means of BMI and lung function variables between BMI categories were done by independent T-test, and across quartiles of ΔBMI/y by ANOVA. p-values <0.05 were considered statistically significant. Separate linear regression models with each lung function value as dependent variable were constructed including ΔBMI/y, sex, age, changes in smoking habits, pack-years, ICS use, occupational GDF exposure at follow-up and original cohort as independent variables. Subgroup analyses were performed by additionally separating overweight from obese, by stratifying for sex and also by including only those gaining BMI during follow-up (ΔBMI/y >0).

## Results

### Basic characteristics

In total, 62.5% were women among those with normal weight, compared to 48.5% of the overweight/obese. The overweight/obese were older, while non-smoking was more common in the normal-weight group. Mean pack-years was similar in both BMI groups. ICS use at study entry was uncommon, 11.1% (normal weight) and 13.3% (overweight/obese), while at follow-up it increased to 42.1% and 45.9%, respectively (table 1). Basic characteristics are presented separately for overweight and obese subgroups in supplementary table S1.

**TABLE 1:** Basic characteristics at study entry and follow-up by BMI groups at study entry

		**Normal weight**	**Overweight/obese**	**p-value**
**Subjects n**		485	460	
**Sex, female**		303 (62.5)	223 (48.5)	**<0.001**
**Age at study entry years, mean±sd**		37.7±11.4	43.3±11.2	**<0.001**
**Age at follow-up years, mean±sd**		56.6±12.3	61.4±11.9	**<0.001**
**Overweight/obese at follow-up**		283 (58.4)	442 (96.1)	**<0.001**
**Smoking habits at study entry**				
** **Nonsmoker		238 (49.1)	186 (40.4)	
** **Ex-smoker		113 (23.3)	158 (34.3)	
** **Smoker		134 (27.6)	116 (25.2)	**0.001**
**Smoking habits at follow-up**				
** **Nonsmoker		251 (51.8)	202 (43.9)	
** **Ex-smoker		177 (36.5)	209 (45.4)	
** **Smoker		57 (11.8)	49 (10.7)	**0.019**
**Mean pack-years at follow-up, mean±sd** **^#^**		16.5±14.9	17.6±16.6	0.436
**ICS use at study entry**		54 (11.1)	61 (13.3)	0.318
**ICS use at follow-up**		204 (42.1)	211 (45.9)	0.238

Frequencies presented as n (%) unless otherwise stated. Bold values indicate p<0.05. Normal weight: BMI 18.5–24.9; overweight/obese: BMI ≥25. BMI: body mass index. ICS: inhaled corticosteroids. ^#^: among ever-smokers with: normal weight n=234, overweight/obese n=258.

### BMI and lung function

The overweight/obese had lower FEV_1_ at study entry (pp and mL) and follow-up (pp) than the normal-weight group (table 2). However, subjects with normal weight had a larger ΔBMI/y and tended to have faster (i.e. worse) ΔFEV_1_pp/y than the overweight/obese, while there was no difference in ΔFEV_1_mL/y. When studying the overweight and obese separately, the obese presented the lowest FEV_1_ (pp and mL) at study entry but slower ΔBMI/y and ΔFEV_1_/y (pp and mL) than both the overweight and the normal-weight groups. Similar results were seen regarding FVC at study entry and follow-up, while no significant differences between BMI groups were seen regarding ΔFVC/y (pp or mL) and ΔFEV_1_/FVC/y (table 2, supplementary table S2). Distributions of changes in BMI, FEV_1_, FVC and FEV_1_/FVC among subjects with normal weight and overweight/obesity, respectively, are shown in supplementary figure S2.

**TABLE 2:** BMI and lung function by BMI groups at study entry

	**Normal weight**	**Overweight/obese**	**p-value**
**Subjects n**	485	460
**Years between examinations**	18.8±4.4	18.1±4.3	**0.011**
**BMI at study entry**	22.6±1.7	28.9±3.3	**<0.001**
**BMI at follow-up**	25.9±3.3	31.5±4.9	**<0.001**
**ΔBMI/y**	0.179±0.165	0.149±0.242	**0.025**
**FEV_1_pp at study entry**	90.4±13.7	86.6±13.7	**<0.001**
**FEV_1_pp at follow-up**	88.4±16.6	85.8±15.7	**0.015**
**ΔFEV_1_pp/y**	−0.099±0.571	−0.023±0.678	0.062
**FEV_1_mL at study entry**	3 272±818	3 163±804	**0.040**
**FEV_1_mL at follow-up**	2 749±824	2 651±797	0.061
**ΔFEV_1_mL/y**	−27.3±20.0	−27.7±23.1	0.788
**FVCpp at study entry**	89.0±11.5	85.3±11.8	**<0.001**
**FVCpp at follow-up**	95.5±15.1	91.7±14.9	**<0.001**
**ΔFVCpp/y**	0.377±0.603	0.386±0.785	0.847
**FVCmL at study entry**	4 032±953	3 939±957	0.133
**FVCmL at follow-up**	3849±1057	3 706±1060	**0.038**
**ΔFVCmL/y**	−8.3±27.0	−11.5±36.0	0.119
**FEV_1_/FVC at study entry**	0.812±0.076	0.805±0.074	0.143
**FEV_1_/FVC at follow-up**	0.714±0.093	0.717±0.082	0.643
**ΔFEV_1_/FVC/y**	−0.005±0.004	−0.005±0.004	0.122

Results presented as mean±sd unless otherwise stated. Bold values indicate p<0.05. Normal weight: BMI 18.5–24.9; overweight/obese: BMI ≥25. BMI: body mass index; FEV_1_: forced expiratory volume in 1 s; FVC: forced vital capacity; ΔBMI/y: annual BMI change; ΔFEV_1_pp/y: annual FEV_1_pp decline; ΔFVCpp/y: annual FVCpp decline; pp: % of predicted; ΔFEV_1_mL/y: annual FEV_1_mL decline; ΔFVCmL/y: annual FVCmL decline; ΔFEV_1_/FVC/y: annual FEV_1_/FVC decline.

### Associations between changes in BMI and lung function

**FIGURE 1:**
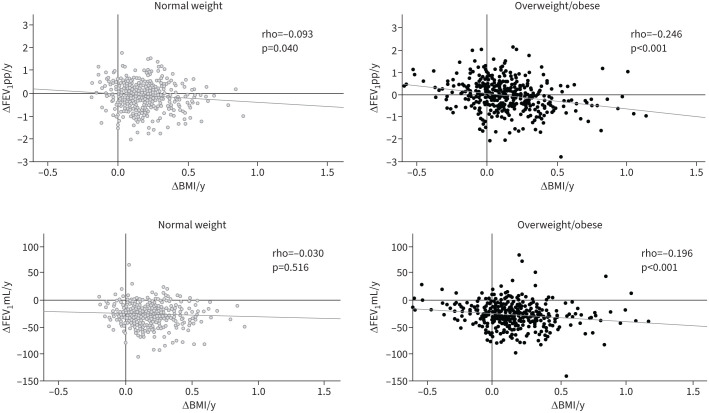
Correlations of ΔBMI/y with ΔFEV_1_pp/y and FEV_1_mL/y by BMI group presented as scatterplots with rho coefficients and p-values. Grey circles: normal weight (BMI 18.5–24.9), n=485; black circles: overweight/obese (BMI ≥25), n=460. BMI: body mass index; y: year; FEV_1_: forced expiratory volume in 1 s; pp: per cent of predicted.

Correlation analysis between annual changes in BMI and FEV_1_ were performed separately among those with normal weight and overweight/obesity. These analyses yielded a rho in the overweight/obese of −0.246 (p<0.001) for ΔFEV_1_pp/y and −0.196 (p<0.001) for ΔFEV_1_mL/y, compared to −0.093 (p=0.040) for ΔFEV_1_pp/y and −0.030 (p=0.516) for ΔFEV_1_mL/y in the normal weight group (figure 1). Correlation analysis of ΔBMI/y with ΔFVCpp/y, ΔFVCmL/y and ΔFEV_1_/FVC/y showed the same pattern as for FEV_1_ with significant stronger negative correlations for FVC (pp and mL) in the overweight/obese. In contrast, ΔFEV_1_/FVC/y had a significant but weak negative correlation with ΔBMI/y among those with normal weight (rho=−0.104, p=0.022) but not among the overweight/obese (rho=−0.026, p=0.572) (supplementary figure S2).

To compare those who gained most BMI and those who gained the least or even decreased their BMI, the sample was divided by quartiles of ΔBMI/y. Among the overweight/obese, a linear trend was seen where the larger the ΔBMI/y (from Q1 to Q4), the faster the ΔFEV_1_/y (pp and mL). No linear trend was seen among those with normal weight (table 3). Similar results were seen for ΔFVC/y (pp and mL), while no linear trends in ΔFEV_1_/FVC/y or pack-years were observed (supplementary table S3).

**TABLE 3:** Mean changes in BMI and changes in lung function within quartiles based on ΔBMI/y, by BMI groups at study entry

	**Quartiles of ΔBMI/y**				
	**Q1**	**Q2**	**Q3**	**Q4**	**p-value**
**Normal weight**					
** **Subjects n	90	140	134	121	
** **ΔBMI/y	−0.024±0.055	0.091±0.029	0.206±0.037	0.402±0.119	**<0.001**
** **ΔFEV_1_pp/y	−0.003±0.577	−0.132±0.535	−0.096±0.589	−0.136±0.584	0.322
** **ΔFEV_1_mL/y	−23.6±20.2	−29.8±18.9	−27.9±18.7	−26.6±21.9	0.127
**Overweight/obese**					
** **Subjects n	146	97	102	115	
** **ΔBMI/y	−0.091±0.137	0.095±0.029	0.195±0.036	0.457±0.193	**<0.001**
** **ΔFEV_1_pp/y	0.125±0.695	0.030±0.625	−0.036±0.674	−0.244±0.651	**<0.001**
** **ΔFEV_1_mL/y	−24.4±21.2	−26.8±20.2	−27.6±26.2	−32.8±24.0	**0.032**

Results presented as mean±sd unless otherwise stated. Bold values indicate p<0.05. p-values are used to test differences in mean values across quartiles of ΔBMI/y by ANOVA. Quartile 1: ΔBMI/y <0.042, Quartile 2: 0.042≤ΔBMI/y<0.142, Quartile 3: 0.142≤ΔBMI/y<0.274, Quartile 4: ΔBMI/y ≥0.274. Normal weight: BMI 18.5–24.9; overweight/obese: BMI ≥25. BMI: body mass index; FEV_1_: forced expiratory volume in 1 s; FVC: forced vital capacity; ΔBMI/y: annual BMI change; ΔFEV_1_pp/y: annual FEV_1_pp decline; ΔFEV_1_mL/y: annual FEV_1_mL decline; pp: per cent of predicted.

### Adjusted associations between changes in BMI and lung function

**FIGURE 2:**
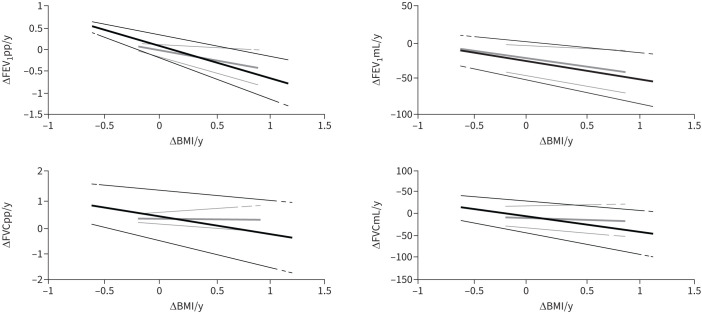
Adjusted regression curves of ΔBMI/y with ΔFEV1pp/y, ΔFEV1mL/y, ΔFVCpp/y and ΔFVCmL/y by BMI groups. Grey lines: normal weight (BMI 18.5–24.9), n=485; black lines: overweight/obese (BMI ≥25), n=460. Regression curves are presented along with 95% confidence intervals. Only significant factors were included in the models for each BMI group (supplementary table S2). BMI: body mass index; FEV_1_: forced expiratory volume in 1 s; FVC: forced vital capacity.

To further analyse the relationship between ΔBMI/y and annual lung function decline, adjusted regression models were constructed. The associations between ΔBMI/y and respectively ΔFEV_1_pp/y and ΔFEV_1_mL/y remained significant after adjustment but were about twice as strong among the overweight/obese compared to those with normal weight. Regarding associations between ΔBMI/y with ΔFVCpp/y and ΔFVCmL/y, significance remained only in the overweight/obese, while no associations were found with ΔFEV_1_/FVC/y (table 4). Adjusted regression curves for associations between ΔBMI/y and lung function outcomes are illustrated in figure 2 (regression coefficients are shown in supplementary table S4).

**TABLE 4:** Association between ΔBMI/y and annual decline in lung function after adjusting for other factors among BMI groups

	**ΔBMI/y**			
	**Normal weight**		**Overweight/obese**	
	**B**	**95% confidence interval**	**B**	**95% confidence interval**
**ΔFEV_1_pp/y**	−0.356	**(−0.663– −0.049)**	−0.747	**(−1.003– −0.491)**
**ΔFEV_1_mL/y**	−14.662	**(−24.903– −4.420)**	−24.809	**(−33.608– −16.010)**
**ΔFVCpp/y**	−0.102	(−0.436–0.232)	−0.719	**(−1.026– −0.413)**
**ΔFVCmL/y**	−10.091	(−23.509–3.328)	−34.422	**(−48.073– −20.772)**
**ΔFEV_1_/FVC/y**	−0.002	(−0.004–0.000)	−0.000	(−0.002–0.002)

Significant values are given in bold. Normal weight: BMI 18.5–24.9; overweight/obese: BMI ≥25. Adjusting factors: sex, age, changes in smoking habits, pack-years, inhaled corticosteroid use, occupational gas, dust or fumes exposure at follow-up and original cohort. BMI: body mass index; FEV_1_: forced expiratory volume in 1 s; FVC: forced vital capacity; B: β-coefficient from linear regression models; ΔBMI/y: annual BMI change; ΔFEV_1_pp/y: annual FEV_1_pp decline; ΔFVCpp/y: annual FVCpp decline; pp: per cent of predicted; ΔFEV_1_mL/y: annual FEV_1_mL decline; ΔFVCmL/y: annual FVCmL decline; ΔFEV_1_/FVC/y: annual FEV_1_/FVC decline.

### Regression analyses in subgroups

As BMI increase was of particular interest, analyses including only subjects with a BMI increase between study entry and follow-up (n=782) were performed. The overweight/obese had strong associations between ΔBMI/y with ΔFEV_1_/y and ΔFVC/y (pp and mL) while no significance was seen in their normal-weight counterparts. No significant associations were seen regarding ΔFEV_1_/FVC/y (table 5).

**TABLE 5:** Association between ΔBMI/y and annual decline in lung function after adjusting for other factors among only BMI gainers by BMI groups

	**ΔBMI/y**			
	**Normal weight (n=432)**		**Overweight/obese (n=350)**	
	**B**	**95% confidence interval**	**B**	**95% confidence interval**
**ΔFEV_1_pp/y**	−0.287	(−0.642–0.069)	−0.731	**(−1.098– −0.364)**
**ΔFEV_1_mL/y**	−10.745	(−22.882–1.392)	−22.437	**(−35.813– −9.062)**
**ΔFVCpp/y**	−0.043	(−0.430–0.343)	−0.718	**(−1.164– −0.271)**
**ΔFVCmL/y**	−4.857	(−20.482–10.767)	−35.251	**(−55.969– −14.533)**
**ΔFEV_1_/FVC/y**	−0.002	(−0.005–0.001)	0.000	(−0.002–0.003)

Significant values in bold. Normal weight: BMI 18.5–24.9; overweight/obese: BMI ≥25. BMI gainer refers to those with ΔBMI/y >0. Adjusting factors: sex, age, changes in smoking habits, pack-years, inhaled corticosteroid use, occupational gas, dust or fumes exposure at follow-up and original cohort. BMI: body mass index; B: β-coefficient from linear regression models; FEV_1_: forced expiratory volume in 1 s; FVC: forced vital capacity; ΔBMI/y: annual BMI change; ΔFEV_1_pp/y: annual FEV_1_pp decline; ΔFVCpp/y: annual FVCpp decline; pp: per cent of predicted; ΔFEV_1_mL/y: annual FEV_1_mL decline; ΔFVCmL/y: annual FVCmL decline; ΔFEV_1_/FVC/y: annual FEV_1_/FVC decline.

Stratified analyses were also performed to see if the observed associations were present in both sexes. All lung function values, now also with the addition of ΔFEV_1_/FVC/y, were associated to ΔBMI/y among both women and men with overweight/obesity (supplementary table S5).

Additionally overweight and obese subjects were studied separately. Here, the adjusted associations between ΔBMI/y with ΔFEV_1_/y and ΔFVC/y (pp and mL) remained significant and stronger compared to the normal-weight group for both subgroups. No associations were seen for ΔFEV_1_/FVC/y (supplementary table S6).

## Discussion

This long-term prospective asthma cohort study highlights BMI increase as a risk factor for a worse decline in both FEV_1_ and FVC particularly among those who are overweight or obese at study entry, independently of ICS use and several other factors. BMI increase associated weakly with the decline in FEV_1_, but not FVC, also in those with normal weight, while BMI change (increase or decrease) did not associate with the decline in FEV_1_/FVC regardless of having normal weight, overweight or obesity at study entry.

A BMI ≥25 is associated with higher incidence and prevalence of asthma [31, 32] and often a more severe clinical presentation among adults with asthma [7, 10, 18]. Lower lung function levels in overweight/obese than in normal-weight adults have been observed, however with a greater difference among those without asthma than among those with asthma [33], probably related to the fact that adults with asthma generally have lower lung function and thus are less likely to have a large further reduction. In our study, overweight and obese subjects were grouped together in the main analyses not only to maintain equal sample size as those with normal weight, but also as it is known that features of silent systemic inflammation as well as other morbidities associated with obesity can be present not only in obese but also in overweight subjects [34]. Whether BMI change affects lung function decline in the long-term among adults with asthma, and whether this association differs between those with overweight/obesity and those with normal weight, has however not been studied before [25]. Our study enabled such analyses and revealed that BMI increase associates stronger with faster decline in both FEV_1_ and FVC among those who were overweight or obese than among those who were normal weight at study entry, which are novel results. Interestingly, when overweight and obese subjects were studied separately, the association between BMI change and lung function decline tended to be equally strong or even stronger for the overweight subgroup compared to the obese. Thus, although there is emerging evidence that overweight may associate with a more favourable asthma prognosis in terms of less mortality compared to normal weight [35], this was not observed in our study, which assessed prognosis in terms of lung function.

The effect of BMI increase on lung function has been studied prospectively among healthy adults using the 10-year follow-up of the US CARDIA study [14] and the 6-year follow-up of the Humboldt cohort recruited from the general population [36]. These two studies showed that both BMI level at study entry as well as weight gain during follow-up related to changes in lung function. In the CARDIA study, obesity at study entry associated strongly with excess decline in FVC but not FEV_1_, while the Humboldt study showed such associations for decline in both FVC and FEV_1._ Although not stratifying for baseline BMI group, both studies showed that large weight gain during follow-up associated with excess decline in FEV_1_ as well as FVC [14, 36], which is well in line with our results for adults with asthma with an even longer follow-up period.

Regarding the association between changes in BMI and lung function among adults with asthma, there are few prospective studies. Within the ECRHS [15], 638 asthmatic patients were followed from 1998 to 2002 and were stratified by having airflow obstruction or not at study entry. In line with our results, they showed that those with normal weight had faster FEV_1_ decline during follow-up than the obese. They also showed that those with largest BMI increase also had the fastest FEV_1_ decline. In contrast to our study, they did not stratify for BMI at study entry and did not perform analysis of association between BMI change and decline in FEV_1_, and further they did not analyse changes in FVC or FEV_1_/FVC [15]. Our study confirmed a strong association between BMI increase and FEV_1_ decline in adults with asthma, but also revealed that this association was twice as strong among the overweight/obese at study entry compared to normal-weight subjects. In addition, regarding FVC the corresponding associations in our study were only seen in those with overweight and obesity at study entry. As no associations between BMI change and FEV_1_/FVC were seen in our study, this implies that BMI increase may mostly relate to the development of a restrictive rather than obstructive lung function pattern.

Asthma is a heterogeneous condition with different phenotypes, including the non-Th2-related (obesity-related and neutrophilic) [16]. The obesity-related asthma phenotype is often complicated with the presence of several obesity-related comorbidities such as diabetes or ischaemic heart disease [17, 18]. It is also known that, for example, obstructive sleep apnoea syndrome and gastro-esophageal reflux, which are common comorbidities particularly in severe asthma [37], may impact the association between BMI and lung function. Smoking also has adverse effects on lung function among adults with asthma [4] and was more common among the overweight/obese than those with normal weight in our study. Further, obesity-related asthma is known more often to be therapy-resistant with regard to corticosteroid use due to less eosinophilic inflammation [19–21]. Obesity also has physiological effects on lung mechanics as the increase in thoracic and abdominal fat tissue increases the compression of the lungs and therefore reduces lung volumes [12, 38]. These premises highlight the potential of weight loss as one of the non-pharmacological treatment alternatives for asthma due to the potential positive long-term effect on lung function in overweight and obese subjects [23, 39].

Limitations of our study include that BMI may not be the most accurate indicator for determining overweight and obesity as it may account poorly for the amount and distribution of fat tissue [40]. However, BMI is widely used, and results are directly applicable to clinical practice. Further, different guidelines and types of spirometers were used at study entry and follow-up resulting in potential systematic differences in lung function measurements between examinations [41]. One example is the implementation of 6 s of forced expiratory time during FVC measuring at follow-up, which can be a possible explanation for why some subjects presented with a higher FVC at follow-up. Still, as this applies for all subjects, the association with change in BMI and the comparability between BMI groups should not be affected.

The long follow-up time may yield a healthy survivor effect both regarding lung function and BMI. This was addressed in previous publications that implied that non-participants at follow-up were more frequently overweight or obese and had lower lung function at study entry than participants. Therefore, subjects with lower lung function and overweight/obesity may be underrepresented at follow-up [26, 42]. This means that the observed associations between changes in BMI and lung function, which in our study seem to be moderate, in fact might be stronger if the healthy survivor effect were not present.

That study entry was between 1986 and 2001 from five different cohorts can be another potential weakness as treatment guidelines changed during the recruitment period. A cohort effect of successively improved lung function in the general population may be present as large-scale studies have presented such evidence [43]. In order to account for this, the regression analyses were adjusted for original cohorts. Further, the exclusion of 61 subjects from the study as they were underweight (BMI <18.5) or lacked valid measurements of BMI or FEV_1_pp at follow-up may reduce the statistical power and could affect the results. However, we prioritised using a complete case method as we could use real measurements and exclusively focus on the normal weight and overweight/obese categories.

Strengths of our study include high participation rate and large sample size which allows for stratification while maintaining a similar number of subjects in both BMI groups. The long follow-up can also be regarded as a strength as there are few prospective studies allowing for 10- to 28-year follow-ups. The large amount of data collected enabled us to adjust for several factors that could act as potential confounders or mediators, *e.g.* smoking habits, which is known to be strongly associated with decreased lung function. Finally, validation studies regarding self-reported physician-diagnosed asthma within OLIN have shown that the positive predictive value is >90% among adults [44].

In conclusion, BMI increase associates with a faster decline in FEV_1_ and FVC but not in FEV_1_/FVC in adults with asthma. The association between BMI change and decline in FEV_1_ and FVC is stronger among those with overweight or obesity in comparison with their normal-weight counterparts. Maintaining or achieving a normal weight should be considered as an important cornerstone in asthma management to avoid excess lung function decline in the long run.

## Supplementary material

10.1183/23120541.00110-2022.Supp1 **Please note:** supplementary material is not edited by the Editorial Office, and is uploaded as it has been supplied by the author.
Supplementary material 00110-2022.SUPPLEMENT
