# Supplementary material for: Body mass index increase: a risk factor for forced expiratory volume in 1 s decline for overweight and obese adults with asthma
Source: ERJ Open Res. 2022 Oct 24;8(4):00110-2022. doi: 10.1183/23120541.00110-2022 (PMC9589325; doi:10.1183/23120541.00110-2022)
Supplement: Supplementary file 1 [file 00110-2022.SUPPLEMENT.pdf]

SUPPLEMENTAL MATERIAL

Supplemental figures

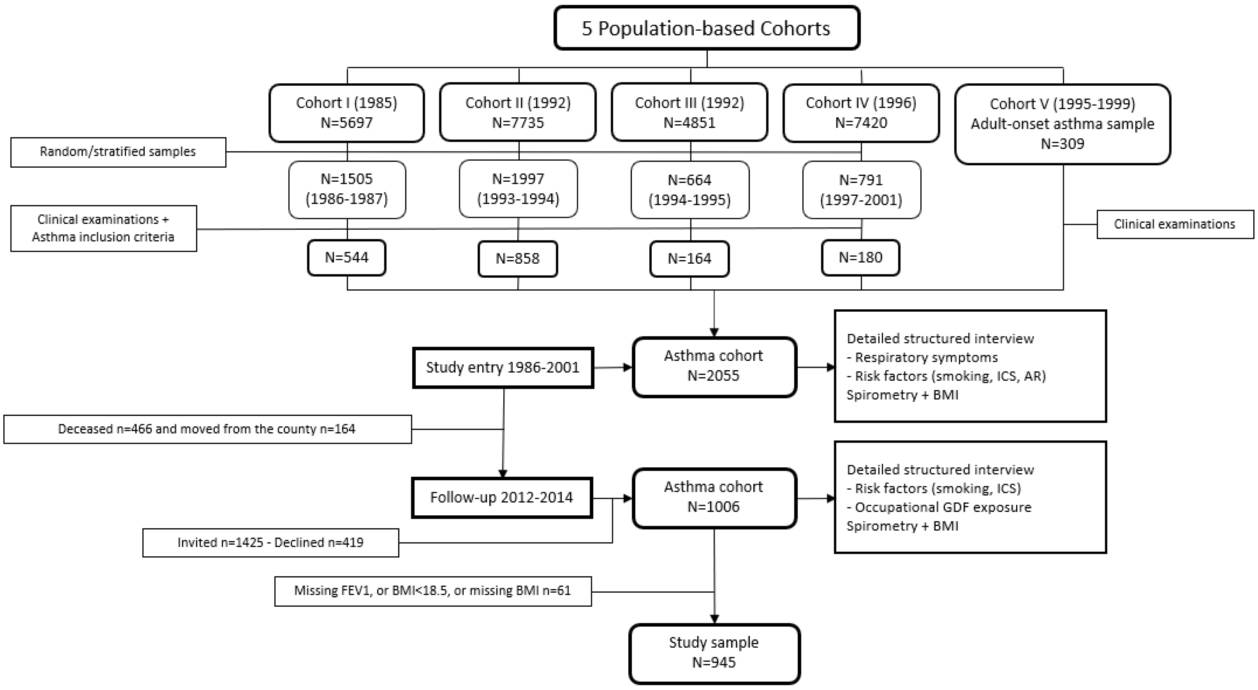

Supplemental figure 1. Study flow chart.

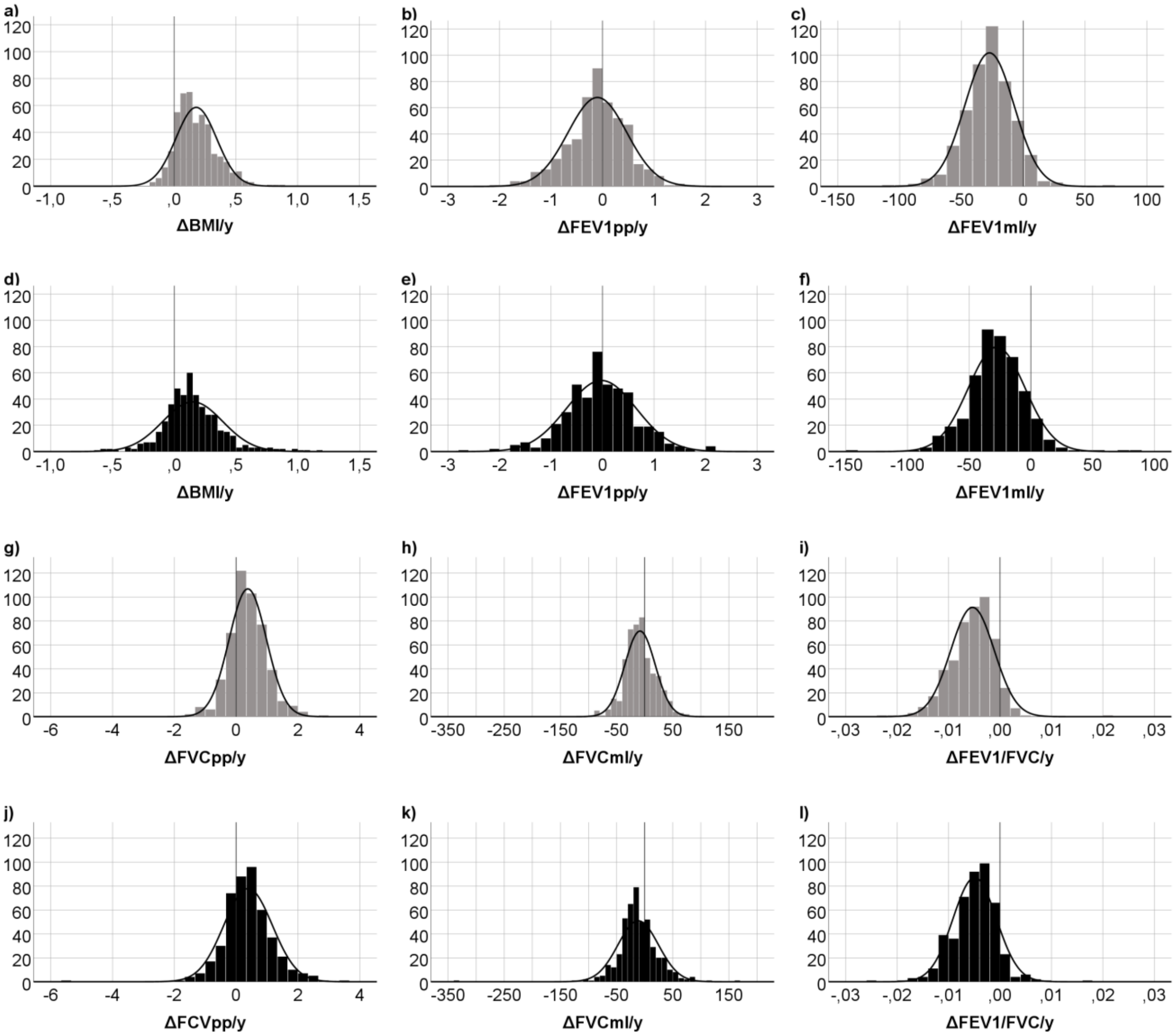

Supplemental figure 2. Distributions of ΔBMI/y, ΔFEV1pp/y, ΔFEV1ml/y, ΔFVCpp/y, ΔFVCml/y and ΔFEV1/FVC/y by BMI group.

Normal weight (BMI=18.5-24.9) n=485. Overweight/obese (BMI≥25) n=460.

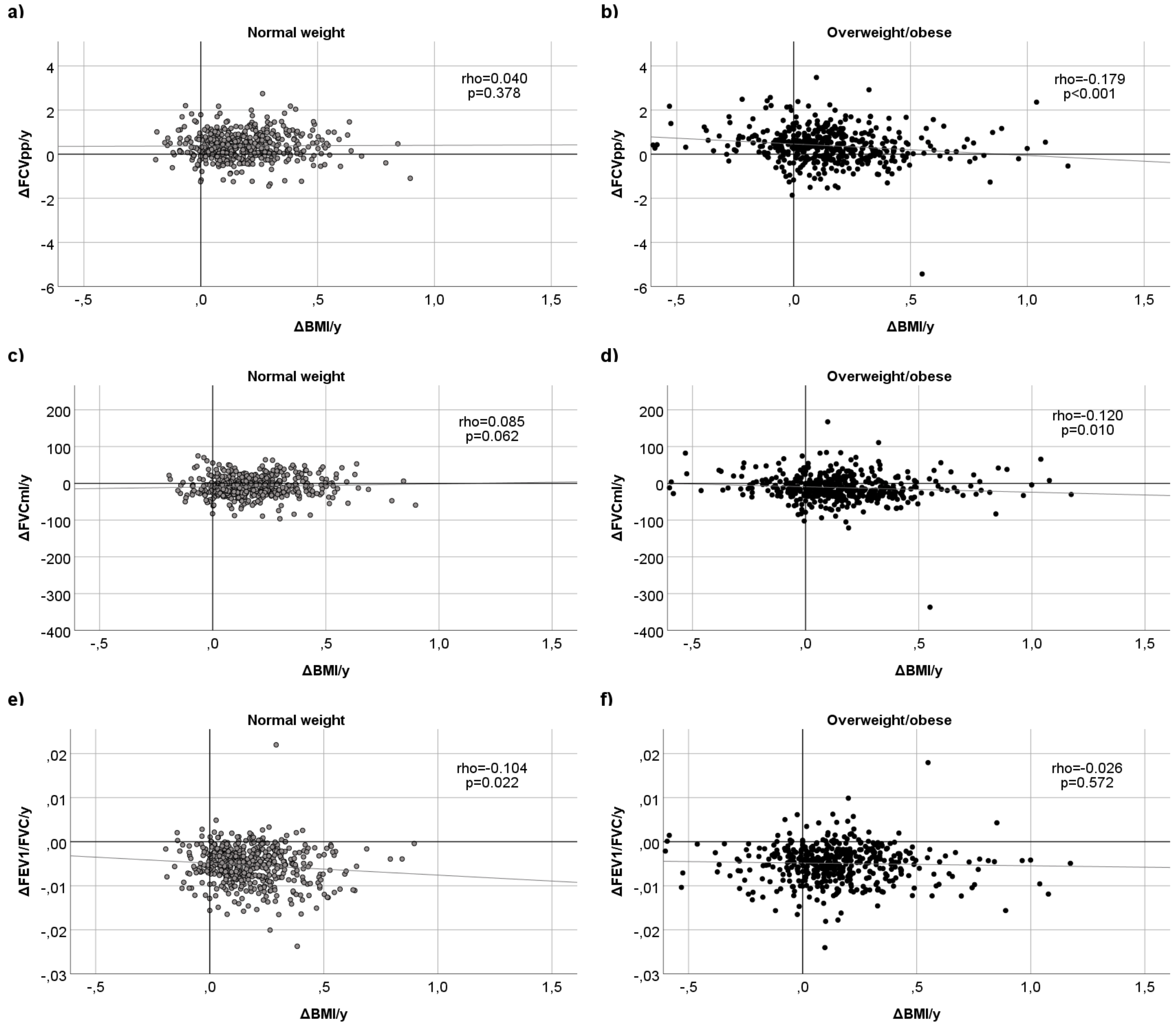

Supplemental figure 3. Correlations of ΔBMI/y with ΔFVCpp/y, ΔFVCml/y and ΔFEV1/FVC/y by BMI group presented as scatterplots with rho coefficients and p-values. ● Normal weight (BMI=18.5-24.9) n=485. ● Overweight/obese (BMI≥25) n=460.

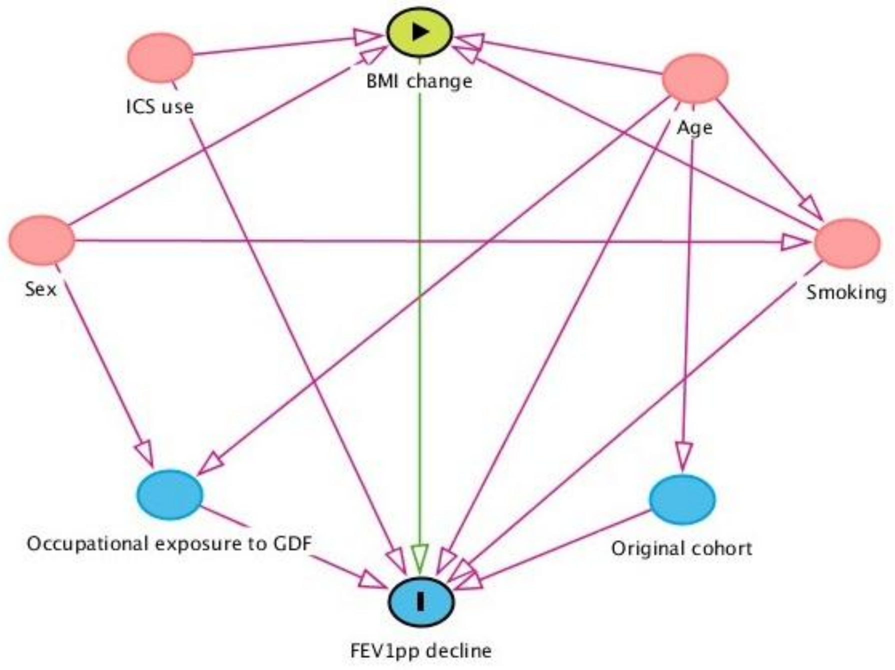

Supplemental figure 4. Directed acyclic graph showing interaction rationale between changes of BMI and lung function with their corresponding related factors.

Supplemental tables

Supplemental table 1. Basic characteristics at study entry and follow-up among overweight and obese groups, with p-values representing comparisons to the normal weight group at study entry

		Overweight/obese n=460					
		Overweight n=327		p-value	Obese n=133		p-value
Sex	Women	145	(44.3)	**<0.001**	78	(58.6)	0.421
Mean age at study entry (SD)		43.3	(11.2)	**<0.001**	43.3	(11.2)	**<0.001**
Mean age at follow-up (SD)		61.6	(11.9)	**<0.001**	61.1	(11.9)	**<0.001**
Smoking habits at study entry	Non-smoker	133	(40.7)		53	(39.8)	
	Ex-smoker	109	(33.3)		49	(36.8)	
	Smoker	85	(26.0)	**0.006**	31	(23.3)	**0.007**
Smoking habits at follow-up	Non-smoker	142	(43.4)		60	(45.1)	
	Ex-smoker	153	(46.8)		56	(42.1)	
	Smoker	32	(9.8)	**0.014**	17	(12.8)	0.391
Mean packyears at follow-up (SD)*		16.6	(14.0)	0.970	20.3	(21.8)	0.170
ICS use at study entry		38	(11.6)	0.830	23	(17.3)	0.057
ICS use at follow-up		145	(44.3)	0.520	66	(49.6)	0.119

Frequencies presented as n(%) unless otherwise stated. SD=Standard deviation.
BMI=Body mass index. Overweight (BMI=25-29.9). Obese (BMI≥30). ICS=Inhaled corticosteroids.
P-values refer to comparison with the normal weight group. Bold values indicate p<0.05.
*Among ever smokers: Overweight n=185. Obese n=73.

Supplemental table 2. BMI and lung function change of overweight and obese groups, with p-values representing comparisons to the normal weight group at study entry

	Elevated BMI n=460					
	Overweight n=327		p-value	Obese n=133		p-value
Years between examinations	18.2	(4.3)	0.064	17.7	(4.2)	**0.011**
BMI at study entry	27.2	(1.4)	**<0.001**	33.0	(3.0)	**<0.001**
BMI at follow-up	30.0	(3.9)	**<0.001**	35.0	(5.4)	**<0.001**
ΔBMI/y	0.161	(0.216)	0.194	0.119	(0.296)	**0.026**
FEV1pp at study entry	87.2	(13.7)	**0.001**	85.1	(13.6)	**<0.001**
FEV1pp at follow-up	85.7	(15.7)	**0.022**	86.0	(15.7)	0.123
ΔFEV1pp/y	-0.055	(0.659)	0.322	0.056	(0.718)	**0.023**
FEV1ml at study entry	3 230	(806)	0.467	3 000	(778)	**0.001**
FEV1ml at follow-up	2 691	(802)	0.314	2 552	(780)	**0.011**
ΔFEV1ml/y	-28.8	(22.4)	0.335	-25.0	(24.5)	0.316
FVCpp at study entry	86.3	(11.7)	**0.002**	82.6	(11.7)	**<0.001**
FVCpp at follow-up	92.1	(14.9)	**0.001**	90.9	(14.7)	**0.001**
ΔFVCpp/y	0.347	(0.797)	0.558	0.482	(0.748)	0.136
FVCml at study entry	4 046	(965)	0.838	3 674	(887)	**<0.001**
FVCml at follow-up	3 783	(1082)	0.387	3 518	(981)	**0.001**
ΔFVCml/y	-13.0	(37.2)	**0.050**	-7.9	(32.7)	0.904
FEV1/FVC at study entry	0.800	(0.077)	**0.035**	0.816	(0.064)	0.568
FEV1/FVC at follow-up	0.714	(0.084)	0.985	0.723	(0.078)	0.235
ΔFEV1/FVC/y	-0.005	(0.004)	0.068	-0.005	(0.004)	0.797

Results presented as Mean (SD). SD=Standard deviation.

P-values refer to comparison with the normal weight group. Bold values indicate p<0.05.

ΔBMI/y=Annual BMI change. BMI=Body mass index. Overweight (BMI=25-29.9). Obese (BMI≥30).

ΔFEV1pp/y=Annual FEV1pp decline. ΔFVCpp/y= Annual FVCpp decline. pp=% of predicted.

ΔFEV1ml/y=Annual FEV1ml decline. ΔFVCml/y= Annual FVCml decline. ml=milliliters.

ΔFEV1/FVC/y= Annual FEV1/FVC decline.

Supplemental table 3. Changes in FVC, FEV_1/FVC and pack-years at follow-up within quartiles based on ΔBMI/y, by BMI groups at study entry

		Quartiles of ΔBMI/y								p-value
Normal weight		Q1 (n=90)		Q2 (n=140)		Q3 (n=134)		Q4 (n=121)		
	ΔFCVpp/y	0.351	(0.616)	0.350	(0.547)	0.391	(0.586)	0.413	(0.677)	0.815
	ΔFVCml/y	-8.5	(29.4)	-11.4	(24.1)	-8.2	(24.7)	-4.5	(30.5)	0.245
	ΔFEV1/FVC/y	-0.005	(0.004)	-0.005	(0.003)	-0.006	(0.004)	-0.006	(0.005)	0.188
	Pack-years*	8.3	(14.9)	7.2	(12.7)	7.6	(13.0)	9.0	(12.8)	0.729
	Pack-years**	19.1	(17.4)	16.3	(14.8)	15.0	(14.9)	16.8	(13.3)	0.597
Overweight/ obese		Q1 (n=146)		Q2 (n=97)		Q3 (n=102)		Q4 (n=115)		
	ΔFCVpp/y	0.515	(0.764)	0.431	(0.734)	0.354	(0.718)	0.213	(0.880)	**0.018**
	ΔFVCml/y	-8.4	(31.5)	-8.6	(35.3)	-13.2	(33.3)	-16.3	(43.3)	0.264
	ΔFEV1/FVC/y	-0.005	(0.004)	-0.005	(0.004)	-0.005	(0.005)	-0.005	(0.004)	0.801
	Pack-years*	12.0	(19.2)	9.9	(13.4)	9.2	(12.3)	7.9	(13.0)	0.171
	Pack-years**	21.1	(21.3)	16.0	(14.0)	16.4	(12.4)	15.6	(14.7)	0.152

Results presented as mean (SD). SD=Standard deviation. Bold values indicate p<0.05.

P-values are used to test differences in mean values across quartiles of ΔBMI/y by ANOVA.

Quartile 1: ΔBMI/y < 0.042, Quartile 2: 0.042 <= ΔBMI/y < 0.142, Quartile 3: 0.142 <= ΔBMI/y < 0.274, Quartile 4: ΔBMI/y => 0.274.

ΔBMI/y=Annual BMI change. BMI=Body mass index. Normal weight (BMI=18.5-24.9). Overweight/obese (BMI≥25).

ΔFVCpp/y= Annual FVCpp decline. ΔFVCml/y= Annual FVCml decline. ΔFEV1/FVC/y= Annual FEV1/FVC decline.

pp=% of predicted. ml=milliliters.

*Among all. **Among ever smokers with: Normal weight n=234. Overweight/obesity n=258.

Supplemental table 4. Regression estimates for ΔBMI/y and other factors significantly associated with lung function outcomes, by BMI groups

		Factor	B	95% Confidence Interval
Normal weight	ΔFEV1pp/y	ΔBMI/y	-0.425	**(-0.719 - -0.131)**
		Sex	-0.259	**(-0.359 - -0.159)**
		Current smokers	-0.389	**(-0.539 - -0.238)**
	ΔFEV1ml/y	ΔBMI/y	-16.451	**(-26.509 - -6.393)**
		Age at follow-up	-0.497	**(-0.635 - -0.359)**
		Sex	-9.622	**(-12.994 - -6.251)**
		Current smokers	-10.549	**(-15.572 - -5.525)**
		Cohort II	-4.866	**(-8.212 - -1.520)**
	ΔFVCpp/y	ΔBMI/y	-0.033	(-0.352 - 0.286)
		Sex	-0.181	**(-0.292 - -0.070)**
		ICS use at follow-up	-0.138	**(-0.247 - -0.030)**
		Cohort IV	0.237	**(0.062 - 0.411)**
		Cohort V	0.194	**(0.056 - 0.333)**
	ΔFVCml/y	ΔBMI/y	-10.089	(-23.315 - 3.136)
		Age at follow-up	-1.006	**(-1.186 - -0.827)**
		Sex	-6.462	**(-10.887 - -2.038)**
		ICS use at follow-up	-4.438	**(-8.779 - -0.097)**
	ΔFEV1/FVC/y	ΔBMI/y	-0.003	**(-0.005 - 0.000)**
		Pack-years	-0.000	**(0.000 - 0.000)**
		Cohort IV	-0.001	(-0.002 - 0.000)
Overweight/obese	ΔFEV1pp/y	ΔBMI/y	-0.708	**(-0.948 - -0.467)**
		Sex	-0.253	**(-0.369 - -0.137)**
		Current smokers	-0.351	**(-0.539 - -0.163)**
		Cohort III	0.191	(-0.002 - 0.383)
		Cohort IV	0.334	**(0.149 - 0.518)**
		Cohort V	0.213	**(0.069 - 0.357)**
	ΔFEV1ml/y	ΔBMI/y	-23.440	**(-32.252 - -14.628)**
		Age at follow-up	-0.482	**(-0.663 - -0.301)**
		Sex	-6.832	**(-10.883 - -2.781)**
	ΔFVCpp/y	ΔBMI/y	-0.714	**(-1.021 - -0.407)**
		Age at follow-up	-0.009	**(-0.015 - -0.002)**
		Current smokers	-0.310	**(-0.540 - -0.080)**
		Cohort IV	0.229	**(0.009 - 0.448)**
		Cohort V	0.171	(-0.002 - 0.345)
	ΔFVCml/y	ΔBMI/y	-34.312	**(-47.825 - -20.800)**
		Age at follow-up	-1.151	**(-1.430 - -0.872)**
		Current smoker	-14.539	**(-24.656 - -4.421)**
	ΔFEV1/FVC/y	ΔBMI/y	-0.001	(-0.002 - 0.001)

B = Beta-coefficient from linear regression models. Significant values in bold.

ΔBMI/y=Annual BMI change. BMI=Body mass index. Normal weight (BMI=18.5-24.9). Overweight/obese (BMI≥25).

ΔFEV1pp/y=Annual FEV1pp decline. ΔFVCpp/y= Annual FVCpp decline. pp=% of predicted.

ΔFEV1ml/y=Annual FEV1ml decline. ΔFVCml/y= Annual FVCml decline. ml=milliliters.

ΔFEV1/FVC/y= Annual FEV1/FVC decline.

Supplemental table 5. Association of lung function and ΔBMI/y after adjusting for other factors among BMI groups at study entry and categorized by sex

	ΔBMI/y			
**Women**	Normal weight n=303		Overweight/obese n=223	
	B	95% Confidence Interval	B	95% Confidence Interval
ΔFEV1pp/y	-0.307	(-0.665 - 0.050)	-0.742	**(-1.053 - -0.430)**
ΔFEV1ml/y	-11.144	**(-21.220 - -1.068)**	-23.923	**(-32.101 - -15.746)**
ΔFCVpp/y	-0.057	(-0.462 - 0.349)	-0.485	**(-0.837 - -0.133)**
ΔFVCml/y	-6.899	(-20.987 - 7.189)	-22.008	**(-33.777 - -10.238)**
ΔFEV1/FVC/y	-0.002	(-0.005 - 0.001)	-0.002	**(-0.004 - 0.000)**
**Men**	Normal weight n=182		Overweight/obese n=223	
	B	95% Confidence Interval	B	95% Confidence Interval
ΔFEV1pp/y	-0.606	(-1.226 - 0.013)	-0.726	**(-1.182 - -0.271)**
ΔFEV1ml/y	-26.504	**(-51.499 - -1.509)**	-25.318	**(-43.844 - -6.792)**
ΔFCVpp/y	-0.349	(-0.967 - 0.269)	-1.151	**(-1.716 - -0.586)**
ΔFVCml/y	-21.846	(-52.417 - 8.725)	-56.669	**(-85.790 - -27.549)**
ΔFEV1/FVC/y	-0.002	(-0.006 - 0.002)	0.003	**(0.000 - 0.007)**

B=Beta-coefficient from linear regression models. Significant values in bold.

ΔBMI/y=Annual BMI change. BMI=Body mass index. Normal weight (BMI=18.5-24.9). Overweight/obese (BMI≥25).

ΔFEV1pp/y=Annual FEV1pp decline. ΔFVCpp/y= Annual FVCpp decline. pp=% of predicted.

ΔFEV1ml/y=Annual FEV1ml decline. ΔFVCml/y= Annual FVCml decline. ml=milliliters.

ΔFEV1/FVC/y= Annual FEV1/FVC decline.

Adjusting factors: Sex, age, changes in smoking habits, pack-years, ICS use, occupational GDF exposure at follow-up and original cohort.

Supplemental table 6. Association between ΔBMI/y and annual decline in lung function after adjusting for other factors of overweight and obese groups

	ΔBMI/y			
	Overweight/obese			
	Overweight n=327		Obese n=133	
	B	95% Confidence Interval	B	95% Confidence Interval
ΔFEV1pp/y	-0.852	**(-1.201 - -0.503)**	-0.670	**(-1.106 - -0.235)**
ΔFEV1ml/y	-25.395	**(-37.506 - -13.283)**	-24.860	**(-39.682 - -10.037)**
ΔFVCpp/y	-0.901	**(-1.338 - -0.464)**	-0.491	**(-0.946 - -0.035)**
ΔFVCml/y	-39.844	**(-59.812 - -19.876)**	-27.047	**(-46.083 - -8.012)**
ΔFEV1/FVC/y	0.000	(-0.002 - 0.003)	-0.001	(-0.004 - 0.001)

B=Beta-coefficient from linear regression models. Significant values in bold.

ΔBMI/y=Annual BMI change. BMI=Body mass index. Overweight (BMI=25-29.9). Obese (BMI≥30).

ΔFEV1pp/y=Annual FEV1pp decline. ΔFVCpp/y= Annual FVCpp decline. pp=% of predicted.

ΔFEV1ml/y=Annual FEV1ml decline. ΔFVCml/y= Annual FVCml decline. ml=milliliters.

ΔFEV1/FVC/y= Annual FEV1/FVC decline.

Adjusting factors: Sex, age, changes in smoking habits, pack-years, ICS use, occupational GDF exposure at follow-up and original cohort.
